# Investigating how nitrogen nutrition and pruning impacts on CBD and THC concentration and plant biomass of *Cannabis sativa*

**DOI:** 10.1038/s41598-023-46369-5

**Published:** 2023-11-09

**Authors:** Enrico Dilena, Dugald C. Close, Ian Hunt, Sandra M. Garland

**Affiliations:** 1 Tasmanian Institute of Agriculture (TIA), University of Tasmania, Life Sciences Building, Level 2, College Rd, Sandy Bay, TAS 7005 Australia; 2 Tasmanian Institute of Agriculture, University of Tasmania, Private Bag 1375, Prospect, TAS 7250 Australia

**Keywords:** Plant sciences, Chemistry

## Abstract

Precise crop fertilization requires an in-depth understanding of plant uptake and utilisation to optimise sustainable production. This study investigated the influence of nitrogen (N) nutrition and pruning on the cannabinoid concentrations and biomass of a commercial cannabis cultivar; the rationale for this study is how N supply and pruning affect cannabinoid yields and concentration in a commercial setting. Clones of a *Cannabis sativa* L. (CBD-type) were grown in a controlled-environment glasshouse in pots with coarse sand. After five weeks of vegetative growth under 210 mg/L N and an 18 h light regime, rates of 30, 60, 210, and 500 mg/L N were applied to plants for twelve weeks and a light regime set at 12 h. Double stem pruning was applied as an additional treatment to investigate efficacy on biomass increase. Biomass, N concentrations, and cannabinoid concentrations were measured after the final harvest. Pruning treatment did not increase cannabinoid concentrations or affect biomass. It was coincidentally found that plants on the glasshouse edge with higher exposure to sunlight developed more biomass and higher cannabinoid concentrations. Only biomass in leaves was increased significantly via higher nitrogen nutrition. Cannabinoid concentration, as well as cannabinoid yield per plant were decreased with the increase in N supply. High rates of fertilizer are not recommended because of reduced cannabinoid concentration and biomass yield: the ideal N supply is likely to be between 60 and 210 mg/L. This research will benefit growers and advisors in understanding the complexity of effects of nitrogen fertiliser and pruning practices on plant biomass and secondary metabolite production in medicinal cannabis.

## Introduction

Nitrogen (N) availability plays a key role in the primary and secondary metabolism of plants. For example in hops (*Humulus lupulus* L., Cannabaceae family) increased rates of N fertiliser (90, 179, and 269 kg N/ha) led to decreased concentrations of the target α-acids and total oil content, while cone yield was not significantly increased at the highest N rate^1^. A study on *Cannabis sativa*^2^ applied 160 mg/L N during the vegetative stage and increasing amounts of N (30, 80, 160, 240, and 320 mg/L) during the flowering stage: the results showed that higher concentration of cannabinoids were obtained at lower N supply with optimal yield at 160 mg/L.

In another study^3^ various N concentrations (0–600 mg/L N) were applied on hemp and cannabinoid concentration and biomass yield were highest at 50 mg/L N.

There is not universal agreement on the precise amount of N that would deliver the highest yield. Indeed, Anderson et al.^3^ applied a wide range of N concentrations (0–600 mg/L N) on hemp, and found that cannabinoid yields were highest at 50–150 mg/L N, and gradually declined with increasing N rates. The authors reported that plant height was progressively stunted with fertilization rate above 50 mg/L N, with clear signs of leaf chlorosis appearing above 450 mg/L N, indicating a nitrogen toxicity response.

It is generally reported that higher N rates result in lower concentrations of cannabinoids^2^. Also James et al.^4^ reported a bell-shaped response curve of CBD and THC with increasing N rates which peaked at 115 and 116 kg/ha, respectively (the range studied was 0–224 kg/ha).

The different optimal range of N supply in those studies might be attributable to N source formulation, pot medium and size, cultivars, frequency and quantity of irrigation, plant density, and/or indoor growing conditions. This study contributes to the growing body of knowledge, using a commercial hemp cultivar. The specific cultivar used in this study was chosen based on its chemistry profile (low THC concentrations and high CBD concentrations) and its promising biomass potential in a commercial breeding program.

Pruning may affect *Cannabis sativa*, but the results in the literature about the effects of pruning on cannabinoid production depend on the particular treatment applied. Danziger and Bernstein^5^ applied many pruning techniques on two drug-type cannabis cultivars (Fuji and Himalaya) and found that 1st branch removal, 2nd branch removal, and double prune treatments had a significant effect on the concentration of some cannabinoids. For example, THCA + THC concentration and biomass were the lowest when 1st branch removal was used while all other pruning treatments had no significant effects in Fuji. In Himalaya, single prune and double prune treatments were associated with a higher THCA + THC concentration but not inflorescence biomass. Pruning generally improved the level of standardization of most cannabinoids by raising the concentrations of the lower parts of the plant^5^. In contrast a higher inflorescence yield was found in the cultivar ‘Topaz’ when the plants were double pruned (i.e. the main stem was removed early in the vegetative stage), keeping the 6 lower branches, followed by a second pruning (removal of 5 cm tip from each of the 6 branches) at the transition-to-flowering stage^5, 6^. Furthermore, other research^7^ found that pruning two industrial-hemp cannabis cultivars increased both CBD concentration and inflorescence biomass. It appears that different cannabis cultivars respond differently to pruning, according to their own genetic potential, although a reduced spatial variability between inflorescences when plants are pruned may be a general phenomenon. The current study applied a “double pruning” technique, with slight variations, in order to stimulate more lateral branching and promote more inflorescence biomass production, as per the most generalisable findings on cannabis pruning^6^.

Where appropriate, this study discusses which ecological theory best explains or is consistent with the results. The growth-differentiation balance hypothesis^8^ seeks to explain plant allocation of secondary metabolites within a species in terms of growth and defence against herbivory imperatives. This plasticity hypothesis is derived from the concept that resource availability alters plant chemical defences for different taxa and classes of compounds^9, 10^. An additional relevant theory is that plant defences can be more concentrated in floral than other organs^11^ as per the Optimal Defence hypothesis^9^. This hypothesis predicts that plant defences are allocated towards tissues and organs that are the most valuable for plant fitness, for example reproductive organs such as inflorescences of cannabis plants.

The aim of commercial producers is to maximize yield and reliably increase profitability: N application typically influences the biomass of medicinal cannabis, yet there are major differences reported in the N range that would beneficially affect cannabinoid concentration, yield, or biomass production^12–18^. The precise aims of this research were to investigate: (1) a wide range of N application rates from deficiency to toxicity using a potassium and calcium-nitrate formulation and; (2), the efficacy of double pruning as a method to induce greater lateral growth and cannabinoid yield.

## Materials and methods

### Plant material, experimental design and growing conditions

Cannabis clones were propagated from cuttings taken from a hemp cultivar mother plant (clone 97, Martha Jane Medical ltd, Australia). This cultivar has typical hemp characteristics, grows to approximately three metres height, with a dominant main stem and female inflorescences rich in CBD but low in THC (< 0.2%). After rooting, the best healthy cuttings (20 in total) were potted in 15 L containers and filled with coarse sand on 23 December 2020. During the first five weeks all plants received uniform fertilization comprised of a standard Hoagland solution^19^. During this period, the plants were cultivated under an 18-h light 6-h dark cycle (18/6 h), under direct sunlight and with the additional use of Sylvania 400W mercury vapour lamps to ensure longer light exposure during vegetative period.

After five weeks of vegetative growth, plants were randomly allocated into four treatment groups of: 30, 60, 210 and 500 mg/mL N (treatment 1, 2, 3, 4 respectively) and the light regime imposed was 12h/12h light/dark. An additional treatment was included: pruning (treatment 5) (this additional treatment received 210 mg/mL N throughout the trial). All treatments had 4 replicates. For the pruning treatment, the top two nodes of the main stem were cut two weeks after potting: this procedure was repeated at the start of the flowering stage when the light regime was set to 12 h and the terminal node from all lateral branches was removed too. The light regime in the glasshouse was re-set to 12/12 h from the time treatments were imposed. Plant density was 1.45 plant/m^2^.

All pots were irrigated by hand with two litres of modified Hoagland’s solution (4 or 5 times a week) and were weekly flushed with 3 L of tap water to prevent accumulation of nutrients in the media. This cultural management was maintained for 12 weeks after treatment initiation until harvest, when more than 50% of the inflorescences of plants showed trichomes with amber colour. The temperature inside the glasshouse ranged between 18-28 °C night/day.

### Nitrogen elemental analysis

The analysis for total N and carbon was determined at the Central Science Laboratory, University of Tasmania, using a Thermo FlashSmart Elemental Analyser (Italy).

Between 0.7 and 1.7 mg of samples were weighed into tin capsules using a Sartorius Cubis II ultra-microbalance (Germany) with an accuracy of ± 0.1 µg. Combustion of the pressed tin cups was achieved in ultra-high purity oxygen at 1000 °C using tungstic oxide on alumina as an oxidising agent followed by reduced copper wires as a reducing agent. The results were calibrated using a certified sulphanilamide standard.

### Plant morphology, biomass and chlorophyll estimation

Plant height, stem diameter, and the number of branches on the main stem were measured during plant growth, starting from the third week after planting and approximately every 2 weeks after that until the end of the experiment. Plant height was measured as the distance from the base of the plant to the top of the main stem. Stem diameter was measured at 3 cm from the plant base with a digital calliper. Biomass of the plant organs was assessed at the termination of the experiment. Biomass (stems, leaves, inflorescences) was weighed after drying for 5 days at 55 °C.

### Cannabinoid analysis

All inflorescences and leaves from each plant were collected, dried and manually crushed until a fine power using a mortar and pestle. A sample of about 10 g was taken and finely ground to a homogenous mixture using a ceramic mortar and pestle. For each sample, about 100 mg of the ground plant material was placed in a 15 mL tube, 5 mL methanol (Sigma-Aldrich) was added, the tube was vortexed (Chiltern Scientific) for 10 min at room temperature and then filtered with a 0.45 μm PVD filter. Cannabinoid concentrations in the filtered plant extracts were analyzed using HPLC (Agilent 1260 Infinity II) which consisted of a quaternary pump, an autosampler, a heated column compartment, and a Diode Array Detector. The detection was carried out in a spectrum mode, at the wavelength range 230 nm to 285 nm. Chromatographic separations were carried out with Agilent 2.7 μm InfinityLab Poroshell 3.0 × 100 mm 12 EC-C18 in methanol:water (0.1% formic acid in water, 0.05% formic acid in methanol), kept at a temperature of 50°C. The injection amount was 3 μL and it was run at a gradient of MeOH:H2O (63:37) to 100% methanol over 20 min at a flow rate of 1.0 mL/min. Calculation of cannabinoid concentrations were based on pure analytical standards: cannabichromene (CBC), cannabichromenic acid (CBCA), cannabigerol (CBG), cannabinol (CBN), cannabigerolic acid (CBGA), cannabidiol (CBD), cannabidiolic acid (CBDA), cannabidivarin (CBDV), tetrahydrocannabivarinic acid (THCVA) and tetrahydrocannabinolic acid (THCA), Δ9-tetrahydrocannabinol (THC), (Cerilliant,Texas, USA). The r^2^ values for linear regressions of the calibrations curves of all cannabinoid standards were > 0.995.

Cannabinoids concentration was calculated in percentage dry weight (grams of cannabinoids/grams of dry material × 100). Total CBD% (CBD% DW + CBDA% DW) was calculated with the following formula which takes into account the difference in molecular weight between CBD and CBDA^20^:

$$CBD_{Total} = CBD \, + \, CBDA \, \times \, 0.877$$

### Statistical analysis and models

Models and correlations for data corresponding to inflorescences, leaves and total (inflorescences plus leaves) measurements were estimated. Outcomes analysed, including cannabinoid concentrations, plant N and biomass were presented as ‘*y*-variables’ with linear regression models that were analogous to ANCOVA set-ups. The ‘*x*-variables’ used in each model were baseline height measurements of the plants before treatment application, a dummy variable created to represent whether or not a plant was on the north- and west-facing window of the glasshouse and a dummy variable for the treatment. Formally, each model structure can be summarised with the following equation:

1 $$y_{ij} = \beta_{0} + \beta_{1} H_{i} + \beta_{2} S_{i} + T_{j} + \varepsilon_{i}$$

in which $y_{ij}$ was the outcome (cannabinoid concentration, plant N or biomass) of plant $i$ (for $i = 1,2, \ldots , 20$) which was in treatment group $j$ (for $j = 1,2, \ldots , 5$), $H_{i}$ was the baseline height of plant $i$ before any treatment was applied, $S_{i}$ was a dummy variable related to the effect of sun on the plants (this “sun-edge” variable was 1 if the plant was on the north-facing or west-facing edge row of the glasshouse, 0 otherwise), $T_{j}$ was the effect of treatment $j$, and $\varepsilon_{i}$ was a random error term which was assumed to be distributed independently and identically Normal (with a mean of zero and constant variance $\sigma^{2}$ for all $i$). The estimated parameters of the model were $\beta_{0}$ (the intercept), $\beta_{1}$ (the baseline covariate effect), $\beta_{2}$ (the ‘sun-edge effect’), $T_{j}$ (the ‘treatment effect’ for treatment $j$) and $\sigma^{2}$.

**Figure 1:**
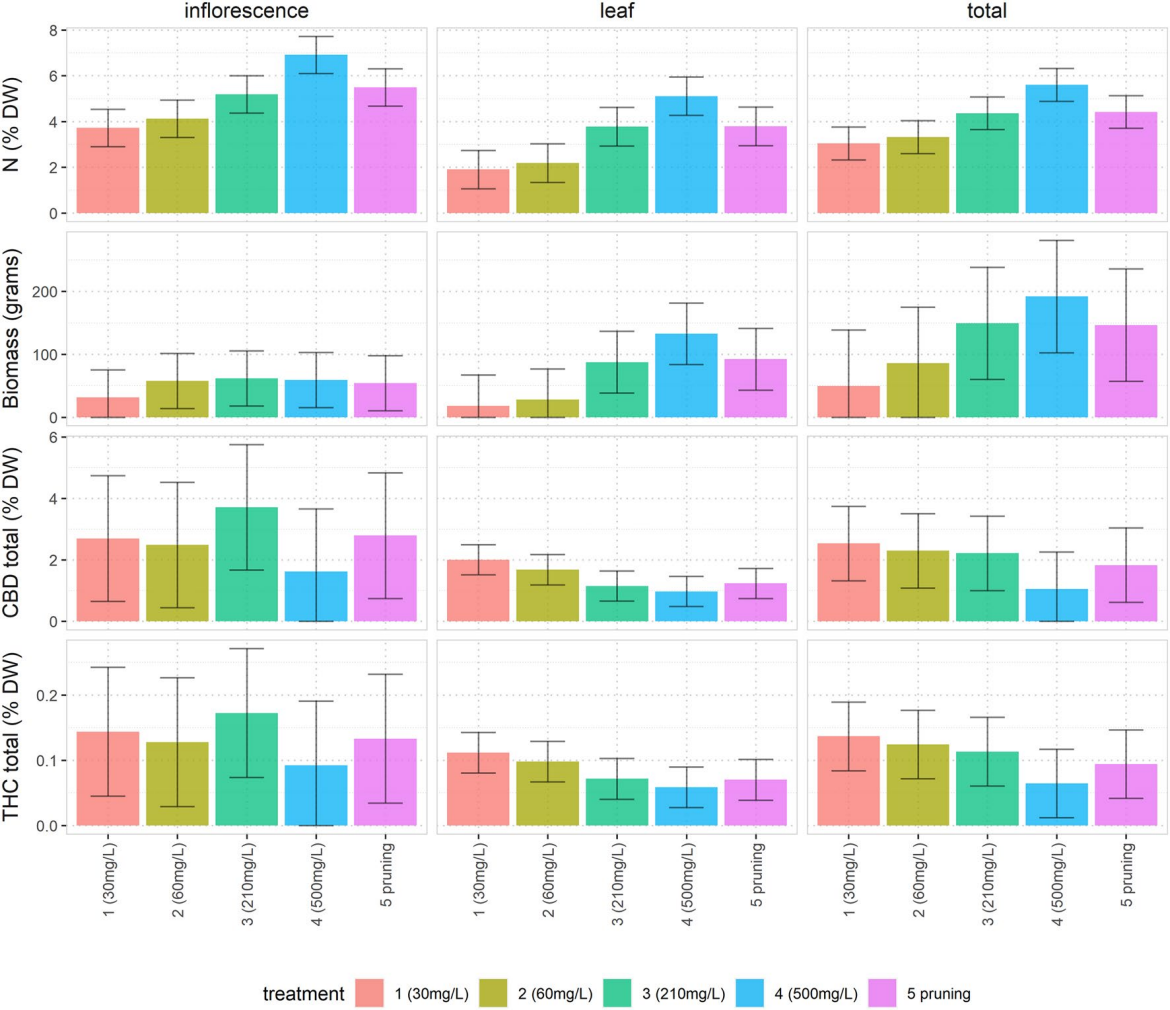
N concentration, biomass, CBD total (CBD + CBDA) and THC total (THC + THCA) in inflorescences, leaves and the total of inflorescences plus leaves (for example, results for the four different measurements on inflorescences are the four bar charts *underneath* the “inflorescence” label at the top of the figure). Each bar (for which n = 4) displays the average level of the outcome variable predicted from the corresponding model based on Eq. (1), with the sun-edge effect removed. The error bars represent the average absolute difference (above or below) between any two treatments that would be notionally “statistically significant” according to a Tukey multiple comparison contrast analysis within the regression model (assuming a Bonferroni adjustment for all pairwise comparisons between the five treatments, using average pairwise standard deviations and a Type I error rate of 0.05). This enables coherent comparisons between treatment effects on an easy to interpret scale. For example, the lower limit of the error bar in the top left chart for treatment 4 does not overlap with the top of the coloured bar for treatment 3 in the same chart: this entails that the p-value is < 0.05 for the test of the null hypothesis that the difference in the effect of treatment 3 and treatment 4 on infloresence N concentration is truly zero. Note: treatment 5 is the pruning stress treatment.

Key model results (Fig. 1) used the average level of outcome variables predicted from the models based on Eq. (1), with the sun-edge effect removed (in practice this entailed estimating the model and issuing predictions for each plant with ${\beta }_{1}$ set to zero). The error bars in the chart represented the average absolute difference (above or below) between any two treatments that would be notionally “statistically significant” according to a Tukey multiple comparison contrast analysis within the model (assuming a Bonferroni adjustment for all pairwise comparisons between the treatments, using average pairwise standard deviations and a Type I error rate of 0.05). This enabled coherent comparisons between treatment effects on an easy to interpret scale. A full set of formal model contrasts and pairwise difference confidence intervals are given in the supplementary material.

In addition to the models implied by Eq. (1), simple linear regressions of outcomes versus plant N concentrations (% DW) were estimated. This approach, using *realised* N concentrations as a numerical variable, compliments the other models that naively use treatment labels as a set of separate categorical variables. These models used the plant N concentrations that were measured in place of treatment dummy variables, and they took the following form:

2 $$y_{ij} = \gamma_{0} + \gamma_{1} N_{i} + \varepsilon_{i}^{*}$$

in which $y_{ij}$ is the outcome (cannabinoid concentration or plant biomass) for plant $i$ (for $i = 1,2, \ldots , 20$), $N_{i}$ was the recorded N concentration for plant $i$ and $\varepsilon_{i}^{*}$ was a random error term which was assumed to be distributed independently and identically Normal (with a mean of zero and constant variance $\sigma_{*}^{2}$ for all $i$). The estimated parameters of the model were $\gamma_{0}$ (the intercept), $\gamma_{1}$ and $\sigma_{*}^{2}$.

Finally, as an auxiliary analysis the relationships between outcomes was examined (cannabinoid concentrations, plant N and biomass) with Pearson correlation coefficients.

The focus of the results analysis was on the categorical contrasts between the estimated treatment effects ${T}_{j}$ in Eq. (1). Overall model fits in terms of adjusted r-squared values and F-test p-values were assessed. The estimated sun-edge effects, ${\beta }_{2}$, from Eq. (1) were also analysed. Equation (2) was used to corroborate the finding in the main results analysis that the effect of N on cannabinoids was both strong and approximately linear. Correlations between outcome variables was cited primarily to justify restricting the presented analysis to a manageable number of outcomes (since many outcomes were highly correlated—see the supplementary tables for all outcome variables).

All analysis was done with R (version 4.1.1), using the base installation packages. P-values were reported to three decimal places (or ‘ < 0.001’, as appropriate).

### Research licence

The research was conducted under a medicinal cannabis licence and permit issued to Martha Jane Medical by the Australian Office of Drug Control and an industrial hemp licence issued to the University of Tasmania under the Tasmanian hemp legislative framework. The plant collection and use were in accordance with all the relevant guidelines.

## Results

### ANOVA results for the treatment effect, sun-edge effect and the baseline height variable

The initial height of plants and exposure to the sun-edge affected the outcome measurements (Table 1). The relative influence of the sun-edge effect on biomass, CBD total concentration (acid + neutral form) and CBD yield in inflorescences and leaves is presented in Table 2. All models presented hereafter were corrected for initial height and sun-edge effects, via the inclusion of each of the variables in Eq. (1).

**Table 1:** Model results for CBD total concentration (total CBD from acid and neutral form).

	Inflorescence		Leaf	
	p-value	Cumulative adj-r^2^ (%)	p-value	Cumulative adj-r^2^ (%)
Baseline height ($\beta_{1}$)	0.127	− 1.1	0.111	− 3.3
Sun-edge ($\beta_{2} )$	< 0.001	50.6	< 0.001	23.4
Treatment ($T_{j}$)	0.034	69.5	< 0.001	86.3

The table presents ANOVA-style results for the linear regression based in Eq. (1) with CBD concentration as the *y*-variable. The inflorescence and leaf columns refer to separate models for each set of data. Each p-value corresponds to a null hypothesis of that true value of the coefficient in that row is equal to zero. The cumulative adj-r^2^ (“adjusted r-squared”) values indicate to what extent the variance of the outcome variable is explained when the model variable in that row is taken into account alongside all variables in higher rows (for example, for the inflorescences model the baseline height and sun-edge effects account for 50.6% of the variance in CBD concentrations). Since the treatment variable is the final model variable to be added, the cumulative adj-r^2^ in that row is the overall model’s adj-r^2^ value. The overall model p-values from an F-test (6 and 13 degrees of freedom) are both < 0.001. For each regression n = 20.

**Table 2:** Total biomass and total cannabinoid concentrations in % DW (on the y-axis) versus measured N concentrations in % DW (on each x-axis). Treatment group labels are indicated by different colours. The shape of the points (circle or triangle) indicates whether or not the plant associated with the data point was on the sun-edge. For each regression n = 20.

	Overall average (includes plants on the sun-edge)	Sun-edge effect (${\beta }_{2}$ from model)	p-value
Inflorescence			
Biomass (grams)	58.5	22.5	0.061
CBD total (% DW)	3.3	2.6	< 0.001
CBD total yield (grams)	2.1	2.5	0.004
Leaf			
Biomass (grams)	74.9	12.9	0.312
CBD total (% DW)	1.5	0.3	0.015
CBD total yield (grams)	1.0	0.3	0.220

The results in Table 1 indicate that the p-values for all coefficients in the inflorescence and leaf models were statistically significant. The practical significance of the variables can be gauged by comparing the relative changes in cumulative adjusted r-squared (adj-r^2^) values as variables were adduced to the regression model: baseline height brought no contribution to the percentage of CBD concentrations explained (*adjusted* r-squared is even slightly negative), but the sun-edge effect and the treatment effects were far more influential (for example, increasing the cumulative adj-r^2^ to 23.4% and 86.3%, respectively, for CBD concentrations in leaves).

A further examination of the sun-edge effect is presented in Table 2, this time for biomass, and CBD total concentrations and yields, for both leaf and inflorescence data. This table presents the overall average (including plants on the sun-edge) of the outcomes, alongside the estimated sun-edge effect from the corresponding model, so that the *scale* of the sun-edge effect is clear (which is a less abstract measure than the contributions to cumulative r-squared made by the sun-edge effect that were presented in Table 1). The sun-edge effects are estimated using models based on Eq. (1): clearly, being on a sun-edge has a significant positive effect on CBD concentration (total CBD from acid and neutral form) (Table 2).

### N and carbon concentrations

Figure 1 displays the average N concentrations in % DW per treatment (based on the estimated models, with the sun-edge effect set to zero) for inflorescences, leaves and overall (inflorescence plus leaf). There is a clear upward trend of N% in inflorescences and leaves from treatment 1 through to treament 4 (recalling that treatments 1–4 have mg/mL N of 30, 60, 210 and 500 respectively). The pruning-stress treatment (5) with 210 mg/mL N applied, had comparable N levels to treatment 3, which was the control group.

There are two important contrasts to make from the N concentration results in Fig. 1. First, treatments 3, and 5 have approximately the same N levels in % DW (5.2, and 5.5 for inflorescences; and 3.8, and 3.8 for leaves) and are not statistically different from each other (as evidenced by the error bars for these treament groups overlapping each others’ estimated average levels). Secondly, compared with all other treatments, the estimated average N concentration level for treatment 4 (6.9 for inflorescences and 5.2 for leaves) is significantly different in a practical and statistical sense.

Carbon concentration has a − 0.90 correlation with N concentration, so there was a clear negative correlation to N concentration (see supplementary material). Relative differences between treatments for carbon levels are therefore analogous to N levels.

### Biomass profile

The biomass results, presented in Fig. 1, differ between inflorescences and leaves. The biomass production for leaves followed a similar pattern to the N concentration—biomass increased in step with the increasing levels of N applied across treatments. But for inflorescences taken separately, there is no practical or statistically significant difference between biomass results for the treatments. For leaves there are apparent differences, but treatment 5 biomass results were very similar to those from treatment 3.

### Cannabinoid concentrations

Cannabinoid results, split by inflorescences, leaves and total, for CBD total and THC total concentrations are summarised in Fig. 1. CBD total (CBD and CBDA) and THC total (THC and THCA) raw values were highly correlated, with a correlation coefficient of 0.98 (p-value < 0.001, Table 4).

There was a clear inverse trend apparent in cannabinoid results for leaves: CBD and THC concentrations decreased steadily between treatments 1, 2, 3 and 4. Furthermore, there was little difference between leaf CBD and THC concentrations for treatment 5, relative to treatment 3.

The inflorescence results for CBD and THC concentrations showed little practical and statistically significant difference between treatments 1, 2, 3 and 5. But there was evidence that treatment 4 had lower concentrations of CBD and THC.

In terms of strict statistical significance for leaves and total CBD and THC concentrations, treatment 4 was different from treatments 1 and 2 (as evidenced by error bars for treatment 4 in Fig. 1 that did not overlap with the estimated average concentrations for treatments 1 and 2). Whilst there was no pairwise statistical significance between treatment 4 and treatment 3, the downward trend in the charts (as N increases) and background information about the effect of N on plants, suggests that the data were in fact consistent with lower total CBD and THC concentrations for treatment 4 (which has N levels of 500 mg/mL N). This inference is corroborated in section “Cannabinoid concentrations”.

Inflorescences CBD yield (in treatment 3) was calculated to be 2.28 g per plant or 3.35 g per metre square.

### Relationship between cannabinoids and plant N levels

**Figure 2:**
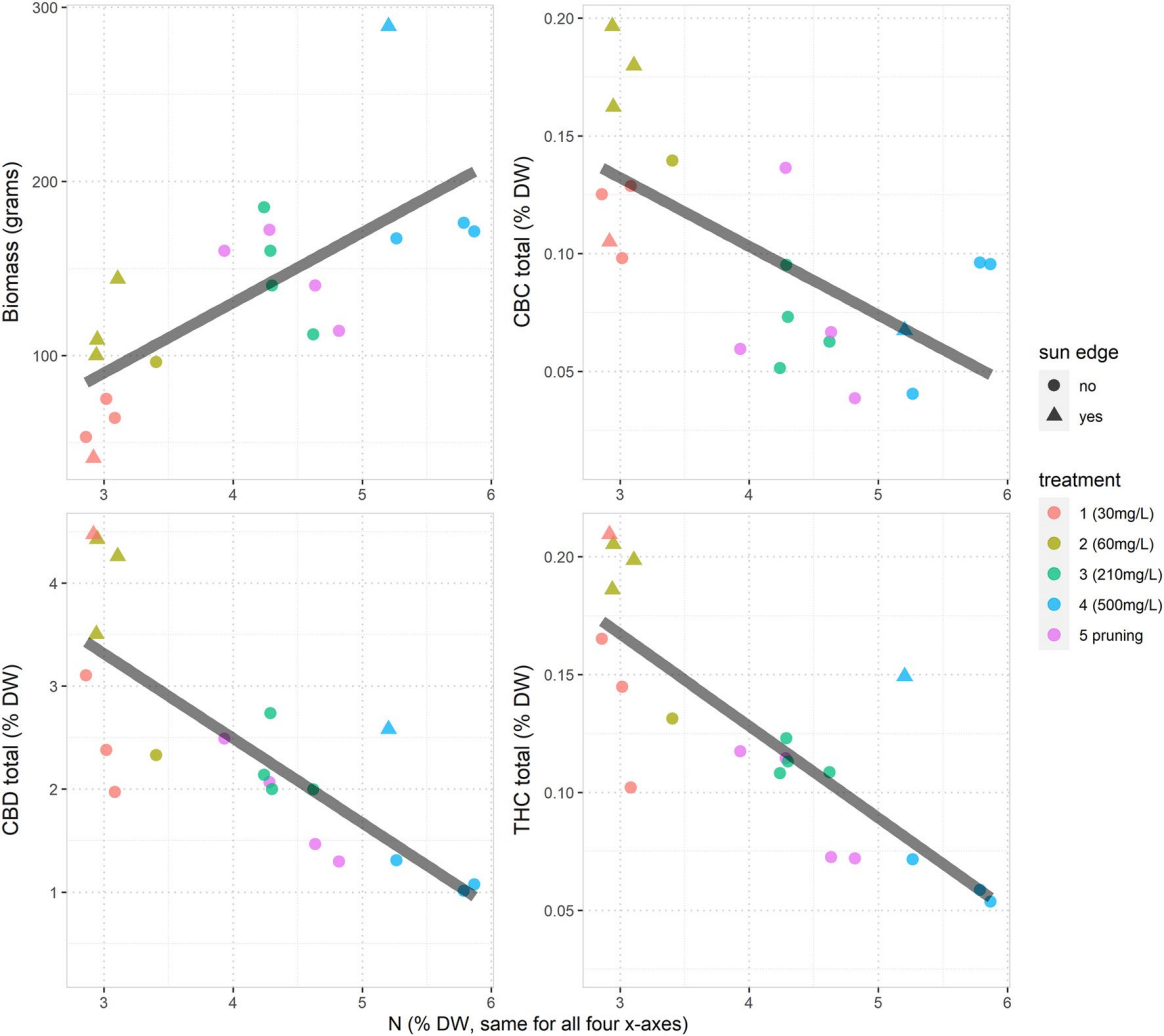
Total biomass and total cannabinoid concentrations in % DW (on the y-axis) versus measured N concentrations in % DW (on each x-axis). Treatment group labels are indicated by different colours. The shape of the points (circle or triangle) indicates whether or not the plant associated with the data point was on the sun-edge. For each regression n = 20.

To further clarify the relationship between N, cannabinoids and yield, in this section the treatment labels were dropped and the modelling re-focused on the relationship between realised N concentrations (% DW) and biomass, CBC total, CBD total and THC total. The linear regression models based on Eq. (2) were estimated, which used N concentration as an x-variable and the other three outcomes as y-variables. The results in Fig. 2 and Table 3 summarise these models and display straight lines according to the predictions from the estimated models.

**Table 3:** Regression summaries for models estimated according to Eq. (2), for CBC total, CBD total, THC total and Biomass as y-variables and N concentration as the x-variable.

	Estimate	95% confidence interval	Adjusted r^2^ (%)	p-value
Biomass (grams)	40.33	(20.51, 60.14)	47.6	< 0.001
CBC total (% DW)	− 0.03	(− 0.05, − 0.01)	37.6	0.002
CBD total (% DW)	− 0.82	(− 1.15, − 0.49)	58.0	< 0.001
THC total (% DW)	− 0.04	(− 0.05, − 0.02)	62.9	< 0.001

Estimate is the slope related to N on each y-variable. The 95% confidence interval is for this slope. Adjusted r-squared values indicate the percentage of variation explained in an outcome variable that is explained by N concentrations. The p-values apply to the null hypothesis that there is no linear relationship between the outcome variable and N. The predicted values of the outcomes are plotted as straight lines in Fig. 2. For each regression n = 20.

The statistical difference of the output data of plants located on the glasshouse’s north and west-facing edge is also salient in Fig. 2. The triangular points in each graph identify the plants on the north- and west-facing edge of the glasshouse: they were consistently above the regression line and on average had higher values than the dot points in the same graphs, which indicated plants not on the edges.

Figure 2 compliments the results presented elsewhere in the following ways. First, biomass was strongly and positively related to N. Secondly, cannabinoid concentrations were strongly and negatively related to N. Thirdly, plants on the sun-edge tended to have higher biomass levels and cannabinoid concentrations.

The most interesting feature of Fig. 2 relates to the scale of the y-axis in each chart: the increase in biomass as N concentration increased from 3 to 6 was approximately 100%; but the corresponding decrease in cannabinoid concentrations was approximately 67% (which entails a net decline in yield in terms of grams as N concentration increases).

### Correlations

CBDA was the cannabinoid with the highest concentration levels detected in the samples and comprised the bulk of CBD total levels (the overall mean concentrations of cannabinoids in inflorescence were: CBDVA 0.17%, CBD 0.21%. CBDA 3.5%, CBGA 0.08%, CBC 0.19%, THC total 0.16%; for leaf, mean concentrations were: CBDVA 0.04%, CBD 0.11%. CBDA 1.5%, CBGA 0.04%, CBC 0.09%, THC total 0.08%). All cannabinoids analysed (including CBCA, THCA, CBDVA) were highly correlated with each other (Table 4). In particular, CBD total (CBD and CBDA) was highly correlated with THC total, CBGA and CBDVA with a Pearson correlation coefficient greater than 0.95. Conversely, as already discussed in the previous sections, CBD total concentration had an inverse correlation to N concentration, so a higher N concentration translated into a lower CBD concentration. CBDA accounted for more than 90% of total CBD.

**Table 4:** Pairwise correlation (Pearson) estimates for overall concentration (% DW) data (inflorescence plus leaf).

	Correlation	p-value
THC total vs CBD total	0.98	< 0.001
CBGA vs CBD total	0.96	< 0.001
CBDVA vs CBD total	0.99	< 0.001
CBC total vs CBD total	0.64	0.003
Carbon vs CBD total	0.81	< 0.001
N vs CBD total	− 0.78	< 0.001

The p-values correspond to the test of a null hypothesis that the true correlation is zero.

### Discussion

The first finding in this study is that higher nitrogen nutrition levels increased biomass and N concentrations (in dry weight percentage, DW%) but tended to decrease cannabinoid concentrations. This result is consistent with the growth differentiation hypothesis that states that when resources are plentiful, growth (e.g., cell division, biomass production), is favoured over differentiation (e.g. cell maturation and production of defensive compounds^21^). The result is important for cannabis crop yields; for example, when N concentration increased from 3 to 6 DW%, biomass increased by approximately 100%, but cannabinoid concentrations decreased by approximately 67%, causing a significant loss in overall yield. This loss in yield is consistent with Anderson et al.^3^, where the effects of various N concentrations (0–600 mg/mL N) on hemp cannabinoid yields were highest at 50 mg/mL N, and gradually declined with increasing N rates. However, Anderson et al.^3^ found that both biomass and cannabinoid concentrations decreased as N nutrition increased—in this study biomass increased with N, just at a lower rate than the corresponding decrease in cannabinoid concentrations. A similar trade-off between cannabinoid concentration and inflorescence biomass has already been reported^17^.

There are subtle differences in the responses to N for leaves and inflorescences. For leaves, when N concentration was higher, biomass was significantly increased and cannabinoid concentration was much lower. For inflorescences, the N concentration, biomass and cannabinoid concentrations were less sensitive to the increases in nitrogen nutrition levels compared with leaves; this observation supports the finding of a previous study which reported that above 160 mg/L N plant organs did not increase in response to higher N rate, except for leaves^17^. This observation is also consistent with the optimal defence theory that predicts within-plant allocation of defences towards organs that enhance plant fitness and with the growth-differentiation balance hypothesis which predicts that when N is limited, plants limit growth and direct excess carbon to the synthesis of carbon-based defence compounds^21^. Indeed, at low N supply, this study found that cannabis plants were shorter with less leaves, but cannabinoid concentration was higher (in leaves) or unchanged (in inflorescences).

For very high levels of N nutrition (500 mg/mL), inflorescence cannabinoid concentrations decreased significantly (with little change in biomass) and leaf biomass increased significantly. With higher N supply stem diameter also increased. This response was found also in other species^22^. This response aligns with the growth-differentiation balance hypothesis^21^: high nutrient availability resulted in lower cannabinoid concentration because resources were allocated more towards growth instead of differentiation. When N supply is restricted, chlorophyll concentration^23^, photosynthesis, and leaf growth^24^ are reduced: indeed, in this study, SPAD chlorophyll estimates were lower in the low N range (see supplementary information). A similar phenomenon was reported when *Brassica carinata* and *Brassica napus* species were subjected to reduced nutrition^25^: N was redirected from leaves to reproductive components, such as inflorescences, while reducing plant height or leaf area. This phenomenon was observed in this study for the cannabis clones with lower N application.

Co-incident and preliminary findings in this study were that baseline height and sun-edge effects had a significant impact on biomass and plant cannabinoid yield; these effects are of interest per se and were accounted for in order to accurately measure the main treatment effects by using ANCOVA-style models that incorporated baseline height and a sun-edge dummy variable as covariates^26^.The sun-edge effect was an artefact that was necessarily taken into account because it was observed to be a significant difference between plants on the glasshouse edge, directly exposed to more sunlight than the plants behind them. The estimates for the sun-edge effect are of particular interest: plants exposed to the north and west-facing edges of the glasshouse (the experiment is in the southern hemisphere) had significantly higher biomass and cannabinoid concentrations; because of their positions, these plants received higher direct sunlight which likely promoted more photosynthesis and higher accumulation of primary and secondary compounds (N-based and C-based). The influence of light intensity on cannabis has been reported in literature^27^: higher LED light exposure led to higher inflorescence biomass and THC concentrations. Conversely, a different study^28^ reported higher inflorescence biomass and CBD yield as light exposure increased, but with no matching increase in CBD concentration. Other authors^29, 30^ also found that light exposure increased biomass, but not cannabinoid concentrations. Higher density cultivation produced cannabis inflorescences with reduced cannabinoid synthesis in the lower canopy and this was linked to less light penetration^31^.

The complexity of the relationship between light and cannabis secondary metabolite production merits further examination. It can be hypothesized that these different cannabis responses to light may be due to the utilization of different lighting sources, light intensity, as well as light spectrums^32^. We caution that the findings of the sun-edge effect in this study were coincidental and therefore should only be considered as preliminary results.

A strong correlation between biomass and plant height has been reported in cannabis^3^ but this relationship was not so strong in this study: the contributions of the baseline height variable to the adj-r squared for regressions on cannabinoid concentrations were much smaller than for the sun-edge effect and treatment effects (Table 1).

In this study, the pruning treatment consisted of double pruning, which entailed removing the top two nodes of the main stem and the tip of all lateral branches at the start of the flowering stage. This treatment increased neither the biomass of leaves or inflorescences, nor the respective cannabinoid concentrations. The literature reports a multitude of effects for pruning. For example, CBD concentration was not affected by pruning, but cannabinoid standardization between top and bottom of inflorescences improved, i.e. less spatial variability^20^. On the other hand, another report found differences in cannabinoid concentrations and yield depending on the pruning technique applied^6^; in particular, higher yields were correlated with higher biomass production under a “double-pruning” treatment; they also reported a variation in cannabinoid concentrations that may be linked to light penetration into the plant canopy (which was affected by pruning). Future research into the combined effects of pruning techniques and sunlight is warranted.

For this particular cultivar, CBD% in leaves was about a third of that of the inflorescences but the biomass was up to four-times higher than inflorescences. There is a lot of CBD to be collected from these leaves. If the process is economically viable, extracting cannabinoids from leaves too can increase the return on investment.

The findings of this research paper have certain limitations. Firstly, the sun-edge effects were not planned for and the distribution of plants with the corresponding treatments were not represented equally with respect to the sun-edge effect; yet the sun-edge effect quantified here was an interesting and relevant artefact of the experimental design that was also required to be taken into account to explain the variations in cannabinoid concentration and inflorescence biomass in response to a priori treatments. Secondly, this study looked at the influence of N on only one indicative commercial cultivar. Future trials could include more cultivars and their response to N, P and K. Finally, this study did not look at the influence of N on terpenes.

## Conclusions

In this study, pruning had no positive impact on cannabinoid yield.

Across all treatments, as an artefact of this trial, sun-edge plants that were more directly exposed to sunlight showed a trend towards more biomass and higher cannabinoid concentrations according to the statistical model developed for this study. This coincidental observation warrants further research that is a priori designed to investigate the phenomenon.

In this study, higher nitrogen nutrition raised the concentration of N in both inflorescence and leaf plant matter. The net effect of increasing nitrogen nutrition on the total yield of cannabinoids was negative because the increase in biomass (which was only significant in leaves) was not enough to offset the concomitant decrease in cannabinoid concentrations.

For commercial production of cannabinoids, this research indicates that very high concentrations of fertilizer are not advisable because of lower cannabinoid concentration and yield, and that the optimal N nutrition is likely to be between 60 and 210 mg/L.

Finally, self-shading of plants can limit the production of cannabinoids, so it is important to tailor plant density and light intensity/spectrum to the clonal varieties being grown.

### Supplementary Information

Supplementary Information.

## Data Availability

The raw datasets used during the current study are available from the corresponding author on reasonable request.

## References

[1] Iskra AE (2019). Influence of nitrogen fertility practices on hop cone quality. J. Am. Soc. Brew. Chem..

[2] Song C, Saloner A, Fait A, Bernstein N (2023). Nitrogen deficiency stimulates cannabinoid biosynthesis in medical cannabis plants by inducing a metabolic shift towards production of low-N metabolites. Ind. Crops Prod..

[3] Anderson SLP, Kjelgren RB, Brym Z (2021). Response of essential oil hemp (*Cannabis sativa* L.) growth, biomass, and cannabinoid profiles to varying fertigation rates. PLoS One.

[4] James MS (2023). Hemp yield and cannabinoid concentrations under variable nitrogen and potassium fertilizer rates. Crop Sci..

[5] Danziger N, Bernstein N (2021). Plant architecture manipulation increases cannabinoid standardization in ‘drug-type’ medical cannabis. Ind. Crops Prod..

[6] Danziger N, Bernstein N (2021). Shape matters: Plant architecture affects chemical uniformity in large-size medical Cannabis plants. Plants.

[7] Folina A (2020). Evaluation of the effect of topping on Cannabidiol (CBD) content in two industrial Hemp (*Cannabis sativa* L.) cultivars. Bulletin of University of Agricultural Sciences and Veterinary Medicine Cluj-Napoca. Horticulture.

[8] Loomis, W. In* Proceedings of the American Society for Horticultural Science, Vol. 29* 240–245 (1932).

[9] Stamp N (2003). Out of the quagmire of plant defense hypotheses. Q. Rev. Biol..

[10] Massad TJ, Dyer LA, Vega CG (2012). Costs of defense and a test of the carbon-nutrient balance and growth-differentiation balance hypotheses for two co-occurring classes of plant defense. PLOS ONE.

[11] Cronin G, Hay ME (1996). Within-plant variation in seaweed palatability and chemical defenses: Optimal defense theory versus the growth-differentiation balance hypothesis. Oecologia.

[12] Potter D (2014). A review of the cultivation and processing of cannabis (*Cannabis sativa* L.) for production of prescription medicines in the UK. Drug Test. Anal..

[13] Campiglia E, Radicetti E, Mancinelli R (2017). Plant density and nitrogen fertilization affect agronomic performance of industrial hemp (*Cannabis sativa* L.) in Mediterranean environment. Ind. Crops Prod..

[14] Christoph-Meier VM (1998). Factors influencing the yield and the quality of hemp (*Cannabis sativa* L.) essential oil. J. Int. Hemp Assoc..

[15] De-Prato L (2022). The cannabinoid profile and growth of hemp (*Cannabis sativa* L.) is influenced by tropical daylengths and temperatures, genotype and nitrogen nutrition. Ind. Crops Prod..

[16] Desaulniers-Brousseau VW, Bo-Sen M, Sarah M, Victorio LM (2021). Cannabinoids and terpenes: How production of photo-protectants can be manipulated to enhance *Cannabis sativa* L. phytochemistry. Front. Plant Sci..

[17] Saloner A, Bernstein N (2021). Nitrogen supply affects cannabinoid and terpenoid profile in medical cannabis (*Cannabis sativa* L.). Ind. Crops Prod..

[18] Vera C, Malhi S, Phelps S, May W, Johnson E (2010). N, P, and S fertilization effects on industrial hemp in Saskatchewan. Can. J. Plant Sci..

[19] Hoagland DR, Arnon DI (1950). The water culture method for growing plants without soil. California Agric. Exp. Stat. Circ..

[20] Crispim Massuela D, Hartung J, Munz S, Erpenbach F, Graeff-Hönninger S (2022). Impact of harvest time and pruning technique on total CBD concentration and yield of medicinal cannabis. Plants.

[21] Herms DA, Mattson WJ (1992). The dilemma of plants: To grow or defend. Q. Rev. Biol..

[22] Yan Z (2019). Biomass allocation in response to nitrogen and phosphorus availability: Insight from experimental manipulations of *Arabidopsis thaliana*. Front. Plant Sci..

[23] Close DB, Chris-Hovenden M (2001). Cold-induced photoinhibition and foliar pigment dynamics of *Eucalyptus nitens* seedlings during establishment. Funct. Plant Biol..

[24] Kozlowski, T. T. & Pallardy, S. G. *Physiology of Woody Plants (Second Edition)* (eds Theodore T. K. & Stephen G. P.) 87–133 (Academic Press, 1997).

[25] Seepaul RS, Ian M, Marois J, George S, Wright DL (2019). *Brassica carinata *and *Brassica napus* growth, nitrogen use, seed, and oil productivity constrained by post-bolting nitrogen deficiency. Crop Sci..

[26] Senn S (2006). Change from baseline and analysis of covariance revisited. Stat. Med..

[27] Hawley DG, Thomas S, Michael DM (2018). Improving cannabis bud quality and yield with subcanopy lighting. HortScience Horts.

[28] Rodriguez-Morrison VL, David ZY (2021). Cannabis yield, potency, and leaf photosynthesis respond differently to increasing light levels in an indoor environment. Front. Plant Sci..

[29] Vanhove W, Van Damme P, Meert N (2011). Factors determining yield and quality of illicit indoor cannabis (*Cannabis* spp.) production. Forens. Sci. Int..

[30] Potter DJ, Duncombe P (2012). The effect of electrical lighting power and irradiance on indoor-grown cannabis potency and yield. J. Forens. Sci..

[31] Danziger N, Bernstein N (2022). Too dense or not too dense: Higher planting density reduces cannabinoid uniformity but increases yield/area in drug-type medical cannabis. Front. Plant Sci..

[32] Trancoso I (2022). *Cannabis sativa* L.: Crop management and abiotic factors that affect phytocannabinoid production. Agronomy.

