# Author Correction: Investigating how nitrogen nutrition and pruning impacts on CBD and THC concentration and plant biomass of *Cannabis sativa*

**DOI:** 10.1038/s41598-024-57679-7

**Published:** 2024-03-28

**Authors:** Enrico Dilena, Dugald C. Close, Ian Hunt, Sandra M. Garland

**Affiliations:** 1 Tasmanian Institute of Agriculture (TIA), University of Tasmania, Life Sciences Building, Level 2, College Rd,, Sandy Bay, TAS 7005 Australia; 2 Tasmanian Institute of Agriculture, University of Tasmania, Private Bag 1375, Prospect, TAS 7250 Australia

Correction to: *Scientific Reports* 10.1038/s41598-023-46369-5, published online 09 November 2023

The original version of this Article contained an error in Figure 1 and Figure 2 where the units for treatments 1, 2, 3 and 4 stated in the legend were incorrect.

The original Figures 1 and 2 and their accompanying legends appear below. The original Article has been corrected.

**Figure 1:**
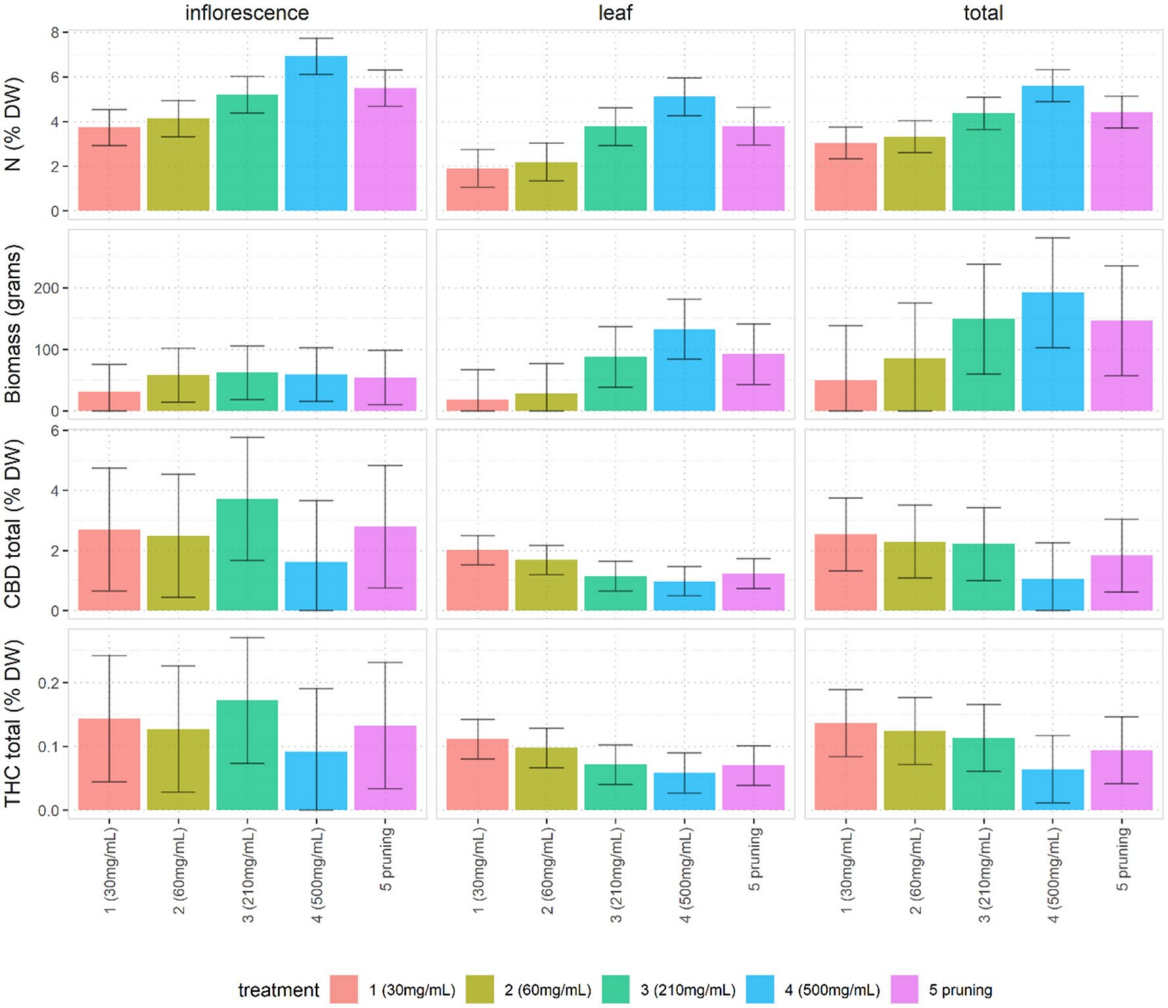
N concentration, biomass, CBD total (CBD + CBDA) and THC total (THC + THCA) in inflorescences, leaves and the total of inflorescences plus leaves (for example, results for the four different measurements on inflorescences are the four bar charts *underneath* the “inflorescence” label at the top of the figure). Each bar (for which n = 4) displays the average level of the outcome variable predicted from the corresponding model based on Eq. (1), with the sun-edge effect removed. The error bars represent the average absolute difference (above or below) between any two treatments that would be notionally “statistically significant” according to a Tukey multiple comparison contrast analysis within the regression model (assuming a Bonferroni adjustment for all pairwise comparisons between the five treatments, using average pairwise standard deviations and a Type I error rate of 0.05). This enables coherent comparisons between treatment effects on an easy to interpret scale. For example, the lower limit of the error bar in the top left chart for treatment 4 does not overlap with the top of the coloured bar for treatment 3 in the same chart: this entails that the p-value is < 0.05 for the test of the null hypothesis that the difference in the effect of treatment 3 and treatment 4 on infloresence N concentration is truly zero. Note: treatment 5 is the pruning stress treatment.

**Figure 2:**
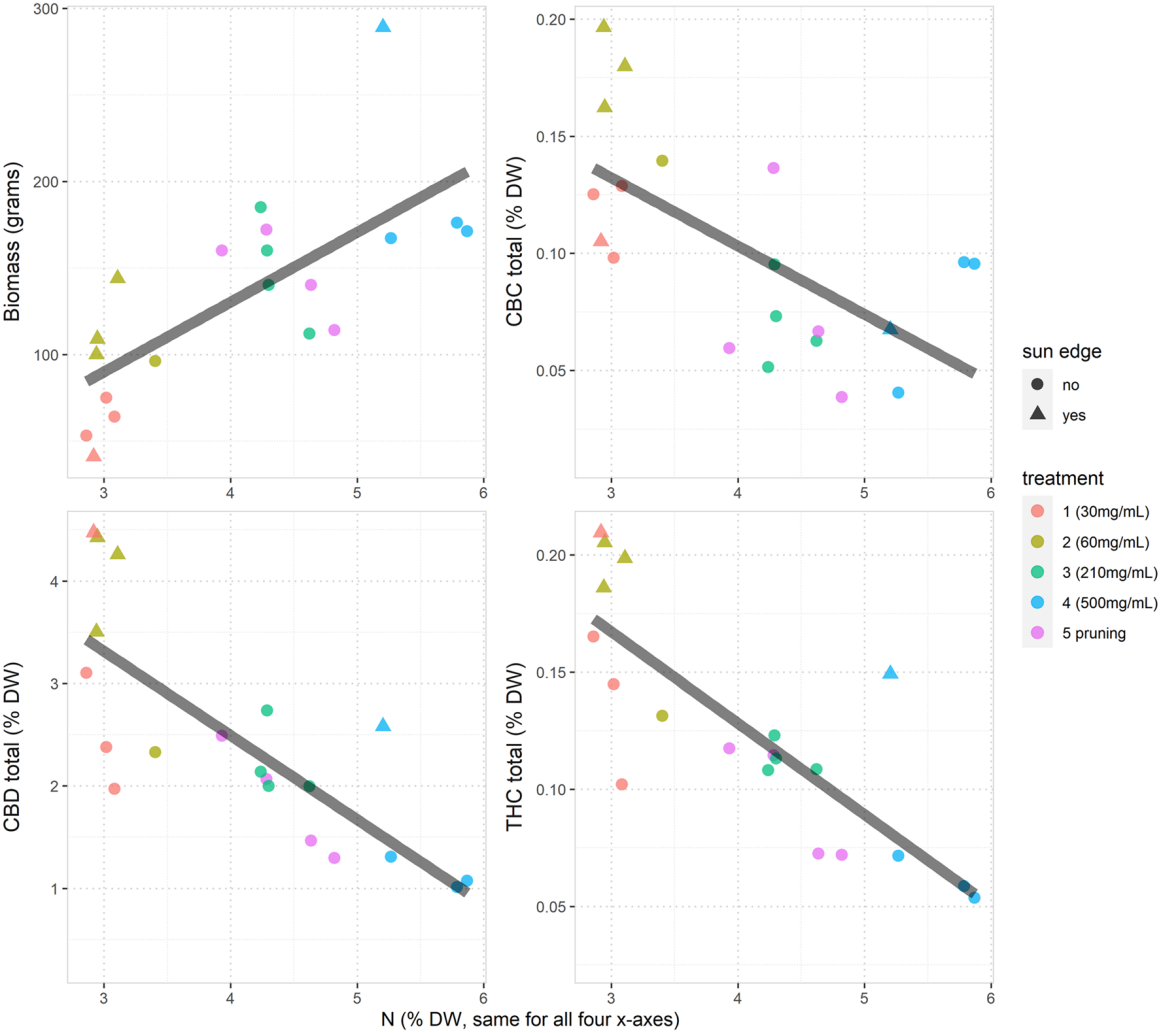
Total biomass and total cannabinoid concentrations in % DW (on the y-axis) versus measured N concentrations in % DW (on each x-axis). Treatment group labels are indicated by different colours. The shape of the points (circle or triangle) indicates whether or not the plant associated with the data point was on the sun-edge. For each regression n = 20.

