# Supplementary material for: Investigating how nitrogen nutrition and pruning impacts on CBD and THC concentration and plant biomass of Cannabis sativa
Source: Sci Rep. 2023 Nov 9;13:19533. doi: 10.1038/s41598-023-46369-5 (PMC10636206; doi:10.1038/s41598-023-46369-5)
Supplement: Supplementary file 1 — Supplementary Information. [file 41598_2023_46369_MOESM1_ESM.docx]

**SUPPLEMENTARY MATERIAL**


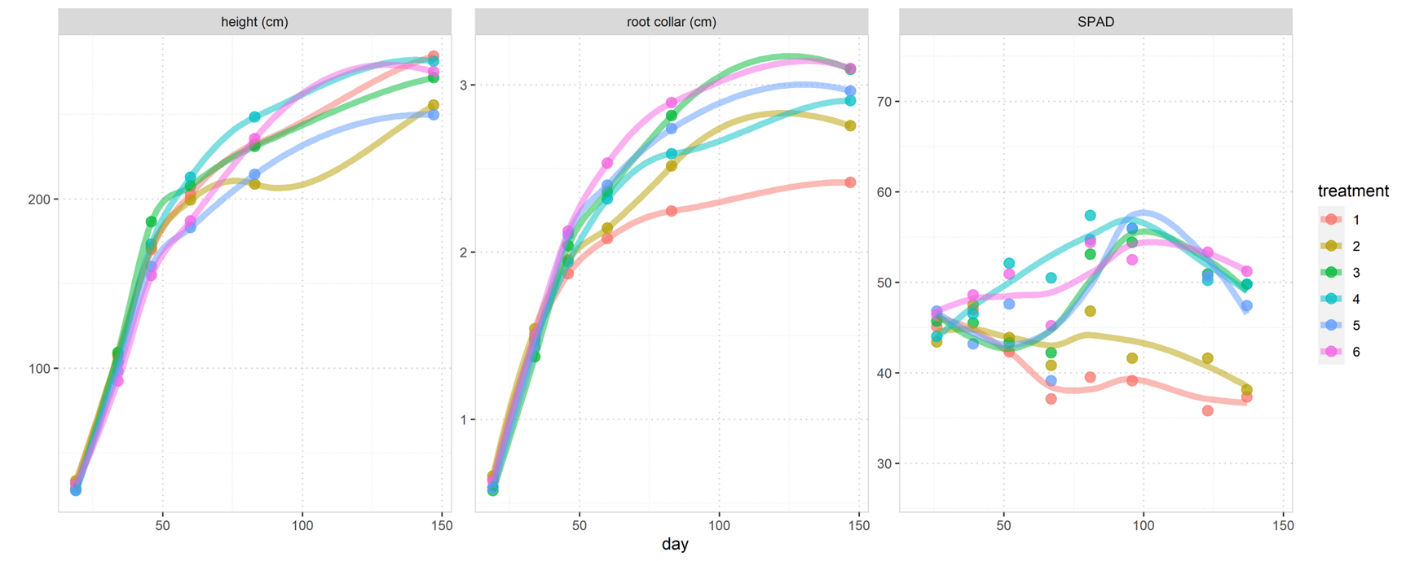


Figure 1S: Cannabis height, stem diameter and chlorophyll estimates (SPAD) until harvest

|  |  | **CBDVA** | **CBD** | **CBDA** | **CBGA** | **CBC_total** | **THC_total** |
| --- | --- | --- | --- | --- | --- | --- | --- |
| **average inflorescence** | | 0.17 | 0.21 | 3.50 | 0.08 | 0.19 | 0.16 |
| **average leaf** | | 0.04 | 0.33 | 1.33 | 0.01 | 0.01 | 0.08 |

Table 1S: average % cannabinoids present in inflorescences and leaves.

| Formal model contrasts from equation (1). | | | | |  |  |  |  |  |
| --- | --- | --- | --- | --- | --- | --- | --- | --- | --- |
| data is for "total" (inflorescence plus leaf) | | | | |  |  |  |  |  |
|  |  |  |  |  |  |  |  |  |  |
| biomass~treatment | |  |  |  |  |  |  |  |  |
|  |  |  |  |  |  |  |  |  |  |
| treatment contrast | coefficients | sigma | tstat | pvalues | pval_bonferroni | df | ci95_low | ci95_high |  |
| 2 minus 1 | 34.91664 | 25.25647 | 1.382483 | 0.190113 | 1 | 13 | -19.6466 | 89.47992 |  |
| 3 minus 1 | 101.7201 | 23.5335 | 4.322356 | 0.000828 | 0.008283 | 13 | 50.87912 | 152.5612 |  |
| 4 minus 1 | 146.4282 | 23.83445 | 6.143553 | 3.52E-05 | 0.000352 | 13 | 94.93701 | 197.9194 |  |
| 5 minus 1 | 90.91733 | 26.44148 | 3.438436 | 0.004405 | 0.044048 | 13 | 33.79399 | 148.0407 |  |
| 3 minus 2 | 66.8035 | 28.00141 | 2.385719 | 0.032954 | 0.329537 | 13 | 6.310133 | 127.2969 |  |
| 4 minus 2 | 111.5116 | 26.02062 | 4.285508 | 0.000887 | 0.008868 | 13 | 55.29744 | 167.7257 |  |
| 5 minus 2 | 56.00069 | 30.72787 | 1.822472 | 0.091458 | 0.914583 | 13 | -10.3828 | 122.3842 |  |
| 4 minus 3 | 44.70806 | 23.99989 | 1.862844 | 0.085231 | 0.852312 | 13 | -7.14056 | 96.55668 |  |
| 5 minus 3 | -10.8028 | 26.7709 | -0.40353 | 0.693119 | 1 | 13 | -68.6378 | 47.0322 |  |
| 5 minus 4 | -55.5109 | 30.19216 | -1.83859 | 0.088925 | 0.889254 | 13 | -120.737 | 9.715328 |  |
|  |  |  |  |  |  |  |  |  |  |
| CBD_total~treatment | | |  |  |  |  |  |  |  |
|  |  |  |  |  |  |  |  |  |  |
| treatment contrast | coefficients | sigma | tstat | pvalues | pval_bonferroni | df | ci95_low | ci95_high |  |
| 2 minus 1 | -0.2181 | 0.343065 | -0.63573 | 0.535981 | 1 | 13 | -0.95924 | 0.523049 |  |
| 3 minus 1 | -0.34692 | 0.319661 | -1.08527 | 0.29752 | 1 | 13 | -1.0375 | 0.343669 |  |
| 4 minus 1 | -1.54668 | 0.323749 | -4.7774 | 0.000361 | 0.003612 | 13 | -2.2461 | -0.84726 |  |
| 5 minus 1 | -0.61371 | 0.359161 | -1.70874 | 0.111248 | 1 | 13 | -1.38964 | 0.162206 |  |
| 3 minus 2 | -0.12882 | 0.38035 | -0.33869 | 0.740254 | 1 | 13 | -0.95052 | 0.692877 |  |
| 4 minus 2 | -1.32858 | 0.353445 | -3.75895 | 0.002386 | 0.023865 | 13 | -2.09215 | -0.56501 |  |
| 5 minus 2 | -0.39562 | 0.417384 | -0.94785 | 0.360495 | 1 | 13 | -1.29732 | 0.506088 |  |
| 4 minus 3 | -1.19976 | 0.325997 | -3.68029 | 0.002772 | 0.027723 | 13 | -1.90403 | -0.49549 |  |
| 5 minus 3 | -0.2668 | 0.363636 | -0.73369 | 0.47616 | 1 | 13 | -1.05238 | 0.51879 |  |
| 5 minus 4 | 0.932964 | 0.410108 | 2.274924 | 0.040498 | 0.404978 | 13 | 0.04698 | 1.818948 |  |
|  |  |  |  |  |  |  |  |  |  |
| THC_total~treatment | | |  |  |  |  |  |  |  |
|  |  |  |  |  |  |  |  |  |  |
| treatment contrast | coefficients | sigma | tstat | pvalues | pval_bonferroni | df | ci95_low | ci95_high |  |
| 2 minus 1 | -0.0126 | 0.014875 | -0.84715 | 0.412236 | 1 | 13 | -0.04474 | 0.019534 |  |
| 3 minus 1 | -0.02333 | 0.01386 | -1.68301 | 0.11622 | 1 | 13 | -0.05327 | 0.006616 |  |
| 4 minus 1 | -0.07191 | 0.014037 | -5.12274 | 0.000196 | 0.001958 | 13 | -0.10223 | -0.04158 |  |
| 5 minus 1 | -0.04292 | 0.015573 | -2.75624 | 0.016342 | 0.163419 | 13 | -0.07656 | -0.00928 |  |
| 3 minus 2 | -0.01073 | 0.016491 | -0.65036 | 0.526787 | 1 | 13 | -0.04635 | 0.024902 |  |
| 4 minus 2 | -0.05931 | 0.015325 | -3.87007 | 0.001933 | 0.019326 | 13 | -0.09242 | -0.0262 |  |
| 5 minus 2 | -0.03032 | 0.018097 | -1.67545 | 0.117717 | 1 | 13 | -0.06942 | 0.008776 |  |
| 4 minus 3 | -0.04858 | 0.014135 | -3.43713 | 0.004416 | 0.044159 | 13 | -0.07912 | -0.01805 |  |
| 5 minus 3 | -0.0196 | 0.015767 | -1.24284 | 0.235877 | 1 | 13 | -0.05366 | 0.014466 |  |
| 5 minus 4 | 0.028987 | 0.017782 | 1.630182 | 0.127041 | 1 | 13 | -0.00943 | 0.067402 |  |
|  |  |  |  |  |  |  |  |  |  |
|  |  |  |  |  |  |  |  |  |  |
| N~treatment | |  |  |  |  |  |  |  |  |
|  |  |  |  |  |  |  |  |  |  |
| treatment contrast | coefficients | sigma | tstat | pvalues | pval_bonferroni | df | ci95_low | ci95_high | p_val |
| 2 minus 1 | 0.280907 | 0.202632 | 1.386291 | 0.188975 | 1 | 13 | -0.15685 | 0.718667 | 0.188975 |
| 3 minus 1 | 1.314399 | 0.188809 | 6.961533 | 9.89E-06 | 9.89E-05 | 13 | 0.906502 | 1.722295 | 9.89E-06 |
| 4 minus 1 | 2.55278 | 0.191223 | 13.34973 | 5.76E-09 | 5.76E-08 | 13 | 2.139667 | 2.965893 | 5.76E-09 |
| 5 minus 1 | 1.386078 | 0.212139 | 6.533804 | 1.90E-05 | 0.00019 | 13 | 0.927778 | 1.844377 | 1.90E-05 |
| 3 minus 2 | 1.033491 | 0.224655 | 4.600353 | 0.000497 | 0.004974 | 13 | 0.548154 | 1.518829 | 0.000497 |
| 4 minus 2 | 2.271873 | 0.208763 | 10.88255 | 6.69E-08 | 6.69E-07 | 13 | 1.820868 | 2.722878 | 6.69E-08 |
| 5 minus 2 | 1.10517 | 0.246529 | 4.48292 | 0.000616 | 0.006163 | 13 | 0.572577 | 1.637764 | 0.000616 |
| 4 minus 3 | 1.238382 | 0.192551 | 6.431457 | 2.23E-05 | 0.000223 | 13 | 0.822401 | 1.654362 | 2.23E-05 |
| 5 minus 3 | 0.071679 | 0.214782 | 0.333729 | 0.743906 | 1 | 13 | -0.39233 | 0.535688 | 0.743906 |
| 5 minus 4 | -1.1667 | 0.242231 | -4.81648 | 0.000337 | 0.003367 | 13 | -1.69001 | -0.64339 | 0.000337 |
|  |  |  |  |  |  |  |  |  |  |

Table 2: pairwise treatment contrasts
